# Knowledge of the signs and symptoms and risk factors of lung cancer in Australia: mixed methods study

**DOI:** 10.1186/s12889-016-3051-8

**Published:** 2016-06-13

**Authors:** Melanie Crane, Nicola Scott, Blythe J. O’Hara, Sanchia Aranda, Mayanne Lafontaine, Ingrid Stacey, Megan Varlow, David Currow

**Affiliations:** School of Public Health, University of Sydney, Sydney, NSW 2006 Australia; Cancer Institute NSW, L9, 8 Central Ave, Australian Technology Park, Eveleigh, NSW 2015 Australia; Cancer Council Australia, L24, 477 Pitt Street, Sydney, NSW 2000 Australia; Faculty of Arts and Social Sciences, University of Technology Sydney, 15 Broadway, Ultimo, NSW 2007 Australia

## Abstract

**Background:**

Lung cancer is the leading cause of cancer death in Australia. There is potential that health promotion about the risks and warning signs of lung cancer could be used to reduce delays in symptom presentation when symptoms are first detected. This study investigated knowledge, attitudes and beliefs which might impact help-seeking behaviour and could provide insight into possible public health interventions in New South Wales (NSW).

**Methods:**

A convergent mixed method study design was used wherein data from 16 qualitative focus groups of residents (40+ years), purposefully recruited and stratified by smoking status, age and geography (metropolitan/regional), were compared with a CATI administered population-wide telephone survey (*n* = 1,000) using the Cancer Research UK cancer awareness measure (LungCAM). Qualitative findings were analysed thematically using NVIVO. Logistic regression analysis was used to investigate predictors of symptom knowledge in STATA. Findings were integrated using triangulation techniques.

**Results:**

Across focus groups, haemoptysis was the only symptom creating a sense of medical urgency. Life experiences evoked a ‘wait and see’ attitude to any health deterioration. Perceived risk was low amongst those at risk with current smokers preferring to deny their risk while former smokers were generally unaware of any ongoing risk. The quantitative sample consisted of females (62 %), 40–65 years (53 %), low SES (53 %), former (46 %) and current smokers (14 %). In quantitative findings, haemoptysis and dyspnoea were the most recognised symptoms across the sample population. Age (<65 years), sex (female) and high socio-economic status contributed to a higher recognition of symptoms. Smoking was recognised as a cause of lung cancer, yet ever-smokers were less likely to recognise the risk of lung cancer due to second-hand smoke (OR 0.7 95 % CI 0.5–0.9).

**Conclusion:**

While there was some recognition of risk factors and symptoms indicative of lung cancer, there was disparity across the sample population. The qualitative findings also suggest that knowledge may not lead to earlier presentation; a lack of urgency about symptoms considered trivial, and smoking-related barriers such as stigma may also contribute to time delays in presentation. Public health interventions may be required to increase awareness of risk and emphasise the importance of seeking medical attention for ongoing symptoms.

## Background

In Australia lung cancer is the leading cause of cancer death for both males and females [1]. Lung cancer survival has improved little over the past thirty years and there are wide international inequalities in survival rates [2]. The five-year relative survival rate for lung cancer is poor at 16 % and in most cases diagnose of lung cancer occurs late, predominantly in Stage III or IV [3]. Earlier detection and diagnosis of lung cancer can lead to improved outcomes in survival rates [4, 5].

One key factor contributing to poorer outcomes for some people with lung cancer is delay in the time it takes to present symptoms from when they are first detected [6–8]. While some people remain asymptomatic until lung cancer is well advanced, others may fail to recognise the seriousness of symptoms experienced, attribute symptoms to existing comorbidity or fail to report changes to their doctor [6, 7, 9]. In addition, people may delay help-seeking due to poor knowledge of lung cancer symptoms, not recognising them as being indicative of a serious disease [10, 11], or wait until their symptoms reach a crisis point [6, 7, 9, 12–14]. Lack of public awareness of lung cancer and recognition of what to do when symptoms develop can contribute to this delay [15].

Evidence also suggests that long-term smoking contributes to delays in the time it takes people to seek medical help [7] and that this may be due in part to fear of blame and the stigma attached to smoking [16, 17] or attributing the symptoms to smoking itself [11]. A synthesis of research into patients’ experiences of recognising symptoms of cancer and help-seeking found that the patient’s gender and the sanctioning of help-seeking were important factors in prompt consultation [12].

While community awareness of the link between smoking and lung cancer in Australia is high [18], awareness of lung cancer symptom recognition is unknown. A number of studies have identified certain symptoms to be more prevalent at various stages of the disease development. For example, symptoms of cough have been reported more frequently in the earlier stages of lung cancer (stage I-III) [7, 19]. Other findings suggest that patients possibly ignore symptoms particularly a persistent cough [7, 9]. It is possible that public knowledge of lung cancer symptoms and therefore ability to recognise symptoms may be higher regarding late stage symptoms such as haemoptysis and less so about symptoms which might appear earlier because of the timing of the symptoms and connection with diagnosis [19].

In New South Wales(NSW), the largest State of Australia, lung cancer is the fourth most common cancer and the leading cause of cancer death [1]. At the State level, health promotion interventions aiming to reduce the incidence of cancer and increase cancer survival are an important component of the NSW Cancer Plan [20]. The primary aim of this study was to comprehensively investigate current knowledge of risk factors and symptoms suggestive of lung cancer in NSW and explore attitudes and beliefs which might impact help-seeking behaviour.

## Methods

### Study design

A convergent parallel mixed-method design utilising quantitative and qualitative data collected in 2012 was used to investigate knowledge, attitudes and beliefs which may influence behaviours in relation to lung cancer symptoms, diagnosis and treatment. The mixed method design provided multiple perspectives on the issue [21].

### Source and study population

Participants in both the qualitative and quantitative study components were male and female, aged over 40 years, resided in metropolitan or regional areas of NSW. NSW is the most populous State of Australia with an estimated population of 7.4 million people. The majority of residents in NSW live in greater Sydney. It is estimated that 50.4 % of the current adult population in NSW has never smoked, while 15.6 % of the population currently smoke [22].

The qualitative research comprised of 16 mixed gender focus groups (7–8 participants) (*n* = 126) (Table 1). Quota sampling was used to select and segment participants by smoking status, age, and socio-economic status (geography-income) (Table 2) as knowledge may differ across these groups. Participants were recruited from a large market research panel where they had opted to be contacted for social marketing research, and were approached via email/telephone. This method allowed for rapid recruitment of a diverse range of people. No prior disclosure of the topic was provided to prevent contamination of participant knowledge.

**Table 1:** Characteristics of the focus groups of NSW residents, Australia, 2012

Location	SES	Age group	Never smoker	Former smoker	Current smoker
Metropolitan	Low	40-64 years	Group 6	Group 12	Group 11
		65+ years		Group 4	Group 3
	Medium-high	40-64 years		Group 1	Group 2
		65+ years			Group 5
Regional	Low	40-64 years	Group 9	Group 8	Group 10
		65+ years			Group 14
	Medium-high	40-64 years		Group 16	Group 13
		65+ years		Group 15	Group 7

Each group included 7-8 participants

**Table 2:** Focus group discussion guide

Discussion question	Theme
What do you know about lung cancer?	Knowledge about lung cancer, diagnosis, treatment, prognosis
What do you think increases someone’s chance of developing lung cancer?	Knowledge & perceptions about causes
Is there something you could do to reduce/prevent lung cancer? What do you think causes lung cancer?	Knowledge & perceptions about causes
What symptoms and warning signs do you think could be associated with lung cancer?	Knowledge & perceptions about sentinel symptoms
What do you think when you hear someone has lung cancer?	Perceived severity and prognosis
Who do you think is most likely to develop lung cancer?	Perceived risk & susceptibility
Do you think that you are at risk of developing lung cancer?	Perceived risk & susceptibility
What would you do if you had symptoms consistent with lung cancer?	Help-seeking behaviours

Participants (*n* = 1,000), in the quantitative study were recruited through a Random Digit Dialling (RDD) sampling frame of landline telephone numbers and selected using the ‘next-birthday’ method where there was more than one eligible member in a household (i.e. the person with the next upcoming birthday was selected where there was more than one eligible person). Quotas were placed on the quantitative sample to include a slightly higher sample of ever smokers (60 %) [23]. This would allow for higher powered sample to investigate relationships within the ever-smoking sample, given the relative risk of lung cancer associated with smoking [24].

### Procedure

#### Qualitative research

Focus groups were used to explore lung cancer awareness of sentinel symptoms and risk factors and beliefs and attitudes. Personal risk, susceptibility and help-seeking behaviours were also explored (Table 2). Focus group discussions were facilitated by three experienced and accredited qualitative interviewers and were semi-structured using a discussion guide (Table 2). Focus groups were conducted face-to-face in community function centre rooms in three metropolitan and three regional locations in NSW. There were no non-participants within the group discussions. Participants provided written consent prior to the focus group discussion. With consent, the metropolitan groups were covertly observed by the authors. Group discussions were conducted for duration of 1.5 h, and participants were reimbursed for their time.

#### Quantitative research

A cross-sectional survey was used to examine lung cancer symptom awareness and help seeking behaviour. The Lung Cancer Awareness Measure (Lung-CAM) was used as the survey instrument. The Lung-CAM is a validated measure developed by University College London and Cancer Research UK which is based on the generic CAM, designed to assess awareness of cancer in the general population [25]. The CAM has been used to monitor awareness over time, compare between groups, identify information needs, and monitor the impact of awareness-raising interventions [26]. Surveys were administered using Computer Assisted Telephone Interviewing (CATI). The cross-cultural face validity of the Lung-CAM was first tested in the Australian population by pilot testing. No issues with the survey’s content or flow were encountered by participants. Participant consent was obtained verbally and recorded. Those conducting the CATI were not aware of the qualitative study findings to reduce the threat of contamination.

Ethical approval was granted by the NSW Population and Health Services Research Committee.

#### Measures/operational definitions

##### Age

Age was split into two groups where possible (40–64 years vs 65+), given the majority of lung cancer cases occur after 65 years the knowledge of symptoms and risk factors may differ as a consequence.

##### Socio-economic status (SES)

Education and income status were combined as one measure. High SES was defined as a household income of more than AUD$80,000 (and any education level), or an income of AUD$40–80,000 and moderate-high education. Moderate SES was defined as an income below AUD$40,000 and high education, or an income of AUD$40–80,000 and moderate education. Low SES was defined as an income below AUD$40,000 and low or moderate education, or an income AUD$40–80,000 and low education. Those with missing data on one variable were classified based on the other.

##### Location-SES

Area postcode was used to determine geographic location as metropolitan (Sydney) or regional/rural location. Postcode was also used as an index of relative socio-economic disadvantage which is a general socio-economic index based on economic and social conditions of people and households within an area [27]. In the qualitative study geographic location was determined by area postcode.

##### Smoking status

Smoking status was defined as “ever smoked daily or weekly” with a subsequent question used to determine current status as a smoker [S], former-smoker [ex-S] or never smoker [NS]. The number of years smoking a pack of cigarettes a day was used as a rudimental measure of pack-years.

##### Lung cancer knowledge

The Lung-CAM comprises of 28 items including knowledge of warning signs (otherwise described as ‘sentinel’ symptoms indicative of lung cancer or symptoms throughout), age of risk, risk factors and behaviour regarding delay in seeking medical help and confidence in detecting symptoms [25]. Two additional questions were added to determine wider help-seeking behaviours including “*who would you go to for help or advice”,* and *“how soon would you discuss it with a family member, friend or partner*” [if you had symptoms you thought might be a sign of lung cancer].

##### Knowledge definition

Knowledge of symptoms and risk factors for lung cancer was defined as correct identification of a sentinel symptom or risk factor (unprompted). Prompted responses were compared with unprompted responses.

##### Knowledge covariates

*‘Previous experience of cancer’* either through personal experience of cancer, or through the experience of close friends/relatives, was included as binary yes/no covariant of knowledge.

##### Help seeking definition

Quantitative participants were asked how confident they were that they would notice a symptom of lung cancer, who would they see if they had a symptom and how soon they would contact their doctor or family/friend to discuss a symptom. Qualitative exploration of what participants would do, who they would see, what symptoms they would seek help for.

### Data collection & quality control

Qualitative and quantitative data were collected concurrently and separately. Focus group discussions were audio recorded and de-identified verbatim transcripts were provided to the researchers for data coding. To assess data quality of the data and coding, we checked data against field notes and checked meanings and nuances with the focus group facilitators to reduce potential bias. We also sought to minimise researcher bias in the coding, ensuring that more than one person contributed to the coding.

The survey took an average of 11.1 min to complete, and a response rate (RR3) of 44 % and cooperation rate (CR3) of 88 % was achieved based on AAPOR eligibility [28]. Quantitative data were collected and weighting applied to adjust for age, sex and regional distribution for population inferences [23]. Post weights were then applied to adjust for differences in the proportion of current smokers weighting to the National Drug Strategy Household Survey, 2010 [29] for population level inferences.

### Analysis

#### Content and thematic analysis

First cycle qualitative data coding was carried out independently by one author and compared with three others who independently coded one third of the data each. A deductive process was first applied to the coding to investigate the predetermined themes. Patterns in the data were then investigated from an inductive approach to explore nuances and emerging constructs within the data. The authors then all met to resolve any discrepancies; validate identified themes and then interpret contextual meaning. Nvivo 10 software (QSR International Pty Ltd. Version 10, 2014) was used to compare themes and models. The credibility of the qualitative data was compared with the literature, particularly findings from a previous study which specifically explored lung cancer knowledge and attitudes in culturally diverse communities in Australia [30]. The data was checked for representativeness across the various demographic groupings. Findings from the content and thematic analyses were then synthesised and variations compared according to theoretical groupings (age, location, socio-economic status and smoking history).

#### Statistical analysis

Pearson’s χ^2^ test was used to test for differences in proportions and t-tests to measure mean differences. To describe demographic variations in lung cancer knowledge, multiple logistic regression modelling was used to analyse recognition of symptoms (with recognition as a binary yes/no variable) based on predictors of lung cancer and demographic variables (smoking history, age, experience of cancer, SES and sex). Symptoms were modelled as follows: symptoms that were recognised by the whole population were modelled in the first model and then stratified according to smoking status and modelled in ever smokers only (this did not include rarer symptoms such as finger clubbing or stridor). Pack years divided at >20 years were included as a predictor in the sub-analysis. In the second set of models only cough symptoms were included as this was felt to reflect the most frequently encountered early symptom of lung cancer by participants and it may give insight into characteristics of awareness at early stages of lung cancer. Sensitivity analysis was used to investigate specific and non-specific symptoms (‘persistent’ ‘painful’ or ‘worsening’ cough verse an unspecified cough). Confidence in recognising symptoms and seeking help for symptoms were also analysed using logistic regression analysis and adjusting for demographic covariates. Statistical analysis was conducted using Stata version 11 (StataCorp LP, College Station, TX).

#### Integration

Data were first analysed separately, and then compared. Equal emphasis was given to both research components [21, 31]. Where possible qualitative data was transformed into count qualities and correlations with the quantitative data investigated. Comparisons were taken between the data sources using quantitative methods and then compared using a qualitative approach. The convergence of themes across the datasets was coded and assessed using triangulation matrices to display and interpret findings [21, 32].

## Results

### Qualitative

#### Knowledge and uncertainty

In exploring participants’ knowledge about lung cancer several themes arose. All groups could identify some sentinel symptoms correctly but there was a great deal of uncertainty and most groups assessed that these symptoms were not necessarily a reason to suspect lung cancer.

*“You can cough up blood and that’s something else, probably”.* -40–55, S, metro

*“You’re guessing about a multitude of things between your mouth and the bottom of your lungs, and there’s any number of things that can make you bleed or cough”. -* 40*–*64, ex-S, regional

There was uncertainty about the relationship between emphysema and lung cancer. Current smokers were particularly confused about the difference, with many believing that lung cancer was a progression from emphysema.

*"Isn't emphysema the start of lung cancer?"* 40–65+, S/Ex-S, metro/regional

#### Help-seeking

Whether knowledge of symptoms consistent with lung cancer would lead to making an appointment with the doctor was discussed. Across all groups, participants mentioned that if they believed symptoms were severe enough (with an ensuing sense of urgency and lack of alternate explanation) then they would go to the doctor, but not because they thought they had lung cancer. Participants qualified this by reporting they know their body, and would know if something was wrong.

*“Life experience teaches us something, you know your own body when you get to our age”.* -65+, ex-S, metro

Haemoptysis and chest pain were the only symptoms mentioned in all groups that would create a sense of urgency. Symptoms such as dyspnoea were considered a normal part of aging. A widely held view was that participants would not discuss with their doctor a symptom that they felt to be ‘mild’, on the assumption that mild symptoms will ‘go away’.

*“No, otherwise you’d be going to the doctor every day of your life”.* - 45–64, NS, metro

#### Barriers to help seeking

Many barriers to help-seeking were reported, including current smokers admitting that they wouldn’t be ‘game’ to go to their doctor about such symptoms because of feelings of stigma associated with smoking. As a result there was a tendency to ‘wait and see’ how bad symptoms become before help-seeking. A sense of fatalism amongst both current and former smokers about their current and future health was also evident.

*“If you know it’s self-inflicted, you’re less likely to go until you really have to”.* - 45–64, ex-S, regional

*“Possibly a feeling that the doctor is going to focus on your smoking “don’t bother coming back until you’ve fixed your smoking”. -* 65+, S, regional

Other barriers to help-seeking included difficulty in accessing a general practitioner (GP), an issue mentioned by all of the regional groups, while the older age groups were more inclined to wait until their next check-up. Fear of bad news was a common barrier across most of the current and former smoker groups, irrespective of age or socio-economic background.

#### Down-playing risk

There was an understanding amongst all participants that smokers were more likely to develop lung cancer. However, there was a strong rejection by smokers that smoking was the only causal factor, with a conviction across the groups of current smokers that *“cancer doesn’t discriminate”.* Smokers down-played the magnitude of the risk due to smoking, providing other causal reasons such as pollution, a stressful job, or blaming ‘newer’ manufactured cigarettes and their additives as opposed to cigarettes smoked in their earlier years.

*“We are so surrounded by many other toxins it’s impossible to isolate cigarettes only”. -* 65+, S, metro

Amongst current smokers, risk susceptibility was down-played. No one felt immune, but dissonant views were held about whether as smokers, they were more at risk than anyone else.

*“We know the facts, yet we still smoke. So we’re obviously in denial about a lot of things. We can accept one point of view and another, because we’re playing mind games with ourselves”.* - 50–64, S, regional

In terms of reducing or balancing risk, smokers felt that by offsetting smoking with other healthy behaviours or smoking less they reduced their risk of lung cancer.

*“By cutting down, eating well and living healthily I’m reducing my risk”.* - 50–64, S, regional

Former smokers generally felt that they were less susceptible to developing lung cancer, with many quoting health messages which seemed to imply that their chance of developing lung cancer would end after quitting smoking.

*“I have a feeling they say 10 years you come back, you got it [the effects of smoking] out of your system”. -* 40*–*64, ex-S, regional

*“They say after three months it’s already clearing”.* - 65+, ex-S, metro

#### Uncertainty about cancer causation

Tied with perception of risk, was a broader uncertainty and limited understanding of cancer. This was unrelated to age, smoking status or socio-economic status. Lung cancer was understood as something that was *“on the bad end of the cancer spectrum”,* though largely confusing, yet something that could be managed through such actions as improving diet and exercise.

*“The basic question is why does cancer strike one person and not someone else? …Am I going to get it? We used to think cancer was catching” -* 65+, ex-S, regional

*“Can they trigger that down or narrow that down to find out what disposition makes someone susceptible to it or any other cancer?”* - 45–64, ex-S, metro

*“I think we all are (susceptible). I think it’s up us to look after ourselves and get as much exercise”* - 45–64, NS, metro

### Quantitative

The majority of survey participants were female (61.2 %); aged >65 years (53.4 %); low socio-economic status (52.9 %); and had a previous experience of cancer, either their own or through a close relative/friend (74.1 %); 14.1 % of the sample currently smoked, 45.9 % were former smokers (Table 3).

**Table 3:** Characteristics of a sample of NSW residents, Australia, 2012 (*n*=1000)

	Number	Percent
	(unweighted)	(weighted)
Sex:		
Female	612	54.4
Male	388	45.6
*Age group:*		
40-64 years	466	67.7
65 years and older	534	32.3
*Index of relative socio-economic disadvantage:*		
1—3 (least disadvantaged)	529	53.5
4—5 (most disadvantaged)	470	46.5
*Education/income combined into socio-economic status:*		
Lowest SES	355	39.2
Intermediate SES	138	18.7
Highest SES	245	42.1
*Location:*		
Regional	400	40.1
Metropolitan	600	59.9
*Smoking status:*		
Current smoker	141	11.8
Former smoker	459	30.5
Never smoked	400	57.8
*Pack years of smoking:*		
Less than or equal to 10 pack years	157	54.0
More than 10 pack years	159	46.0
*Total years of smoking:*		
Less than or equal to 20 years	286	48.9
More than 20 years	309	51.0
*Previous experience of cancer:*		
Self/family/friend with cancer	741	74.5
No close cancer experience	257	25.5

### Knowledge of risk factors

The majority of participants were able to identify smoking as a risk factor for lung cancer (90.6 %, 95 % CI 88.4–92.8), however fewer recognised the risk of second-hand smoke (25.6 %, 95 % CI 22.3–28.9) (Table 4). When compared by smoking status, current smokers were significantly less aware of the risk of lung cancer due to second-hand smoke (AOR 0.4; 95 % CI: 0.2–0.7). This inverse relationship (also observed in the qualitative component) was evident across other risk factors with current smokers more likely to mention factors such as air pollution than never/former smokers (AOR 1.6; 95 % CI: 1.0–2.5).

**Table 4:** Unprompted recognition of risk factors perceived to be associated with lung cancer by NSW residents, Australia, 2012 (*n*=1000) (weighted to NSW population) and triangulation with qualitative focus group data

	Quantitative survey responses					Qualitative focus group responses	
Perceived risk factor for lung cancer	Total (%) 95 % confidence interval	Current smokers (%)	Former smokers (%)	Never smokers (%)	Overall	Count	Comparison
Being a smoker	90.6 (88.4-92.8)	86.6	89.7	91.9	No observed differences between group discussions based on age, geography.	13 groups mention smoking, those that didn’t were all smoker groups	*The link with smoking is very well known and people just ignore that and just keep on coughing away.*
Second-hand smoke	25.6 (22.3-28.9)	14.4**	23.3 *	29.1		7 groups mention passive smoking, those that didn’t were current/exsmoker	*I think certain people are more allergic to nicotine than what otherpeople are.*
Air pollution	21.6 (18.5-24.8)	28.1 *	22.3	20.0	The majority of discussions focused around smoking as a cause and its comparability to other potential factors.	11 groups mention air pollution	*Look if you’re taking anything into your lungs which isn’t clean air, obviously that’s going to have some e effect.*
Chemicals & sprays	24.4 (21.1-27.6)	30.8	23.9	23.3		7 groups mention chemical substances such as poisons, dyes, fertilisers	*Probably the toothpaste has worse chemicals than all of them.*
Asbestos	15.3 (12.6-17.9)	17.3	12.4	16.4	There was confusion about whether it was chance that some people were more susceptible to lung cancer.	8 groups mention pollution	*Like asbestos and that … anything that can affect the lungs and your breathing.*
Occupational exposure	4.9 (3.1-6.7)	2.6	3.9	5.9		8 groups mention occupation	*People working in coal mines could be at risk of lung disease. People working at a chemical plant.*
Hereditary	13.1 (10.8-15.8)	16.9	14.2	12.2		All groups mention a ‘genetic predisposition’	*I do believe that everybody is carrying a cancer gene but it has to have something to activate it.*
Unhealthy lifestyle	14.1 (11.5-16.7)	13.8	12.5	150		Stress, physical inactivity, overweight and unhealthy lifestyles were mentioned by different groups	*Yeah, exactly, and I think if a lot of us paid more attention to our fitness, maybe cancer wouldn’t be quite so common.*
*Diet*	*7.0 (5.0-9.0)*	*5.1*	3.5 *	*9.3*		9 groups believe diet is a cause of lung cancer	*Food and diet additives, preservatives, chemicals in food.*

*p<0.05 **p<0.01 (reference group - never smoker) Factors mentioned but having no known association in italics

### Symptom recognition

The majority of participants correctly identified between one and three symptoms associated with lung cancer (*n* = 820; 82.0 %); 6.5 % identified more than three, while 11.5 % (*n* = 115) were unable to identify any sentinel symptoms of lung cancer. The most recognised symptoms were shortness of breath (55.5 %, 95 % CI 51.9–59.2) and coughing up of blood (39.1 %, 95 % CI 35.4–42.7) (Table 5). Again, these symptoms had also been the most recognised in the focus group discussions. Also, the majority of participants were able to recognise the association between lung cancer and ‘a cough’ more generally (54.8 % 95 % CI 51.1–58.5). No statistically significant differences were observed according to smoking status.

**Table 5:** Unprompted recognition of symptom of lung cancer by NSW residents, Australia, 2012 (*n*=1000) (weighted to NSW population) and triangulation with qualitative focus group data

Quantitative survey responses						Qualitative focus group responses	
						*What are the warning signs of lung cancer?*	
Recognition of symptom	Total (%)	Current smokers (%)	Former smokers (%)	Never smokers (%)	Overall	Count	Comparison
	95% confidence interval						
A painful cough	23.6 (20.4-26.8)	26.5	21.6	24.1	Most understood that these symptoms could be typical of other health conditions, such as respiratory conditions or emphysema	All groups mention cough but the majority were not able to be specific	*There’s cough and there’s cough…when you’re persistently [coughing] it’s a sign of cancer.*
Worsening cough	6.3 (4.5-8.1)	5.3	6.3	6.5			*The kind of cough that comes from way down.*
A persistent cough	17.0 (14.3-19.7)	14.6	19.0	16.5			*A persistent cough, non-stop, sort of thing. It’s been on for days and weeks on end.*
Persistent chest infection	1.9 (0.8-2.9)	0.4	0.6	2.3		Only two mention symptom	*Vulnerable to infection.*
Coughing up blood	39.1 (35.4-42.7)	43.5	37.9	38.8		All groups mention symptom	*You can cough up blood and that’s something else, probably.*
Shortness of breath	55.5 (51.9-59.2)	56.5	54.9	55.7		Most groups mention symptom	*On the ad on telly they say you can hear lung cancer before you can see it.*
Chest pain	10.4 (8.1-12.7)	9.8	10.4	10.4		Most groups mention symptom	*Things in the back of the chest.*
Loss of weight/appetite	3.0 (1.8-4.3)	1.3	3.4	3.2		4 groups mentioned weight loss, only one, mentions appetite	*A dramatic loss of weight.*

In the multivariate analysis, males were significantly less likely to recognise sentinel symptoms as warning signs for lung cancer compared to females (failure to recognise: AOR 2.07; 95 % CI: 1.20–3.59); while adults younger than 65 years were significantly more likely to recognise symptoms (AOR 0.55; 95 % CI: 0.33–0.91) (Table 6). Analysis of all cough related symptoms showed similar findings however age was no longer a significant predictor in the sample population. Participants identified as current or former smokers were no more or less likely to recognise sentinel symptoms. Sub-analysis of these more at risk populations revealed no significant differences in symptom awareness according to pack-years of smoking.

**Table 6:** Logistic regression modelling of symptom recognition of lung cancer on demographic predictors in a sample of residents in NSW, Australia, 2012 (*n*=1000)

					*Failure to recognize any symptom*						*Failure to recognise cough*		
	*All participants*				*Smokers only*			*All participants*			*Smokers only*		
	N	Odds ratio	95 % CI	*p* value	Odds ratio	95 % CI	*p* value	Odds ratio	95 % CI	*p* value	Odds ratio	95 % CI	*p* value
Ag*e group:*													
65 years and older	677	1			1			1			1		
40-64 years	323	0.55	0.33-0.91	0.02	0.27	0.15-0.52	0.000	0.85	0.59-1.21	0.4	0.63	0.40-0.98	0.05
Sex:													
Female	544	1			1			1			1		
Male	456	2.07	1.20-3.59	0.009	1.70	0.93-3.09	0.08	1.59	1.11-2.27	0.01	1.48	0.97-2.27	0.07
*Smoking status (2 categories):*													
Never smoked	578	1			-	-	-	1			-	- -	
Ever smoked daily or weekly	422	0.76	0.45-1.27	0.7				1.04	0.73-1.47	0.8			0.7
*Index of relative socio-economic disadvantage:*													
1—3 (least disadvantaged)	535	1			1			1			1		0.7
4—5 (most disadvantaged)	465	0.87	0.43-1.79	0.7	1.03	0.42-2.52	0.9	0.93	0.59-1.46	0.7	0.89	0.53-1.52	
*Education/income combined into socio economic status:*													
Lowest SES	289	1			1			1			1		
Intermediate SES	138	0.68	0.34-1.42	0.3	0.57	0.26-1.26	0.2	1.46	0.91-2.35	0.1	1.47	0.80-2.71	0.2
Highest SES	311	0.62	0.33-0.19	0.2	0.65	0.29-1.43	0.3	0.99	0.64-1.53	0.9	0.97	0.57-1.66	0.9
*Previous experience of cancer:*													
Self/family/friend with cancer	744	1			1			1			1		
No close cancer experience	255	0.83	0.46-1.50	0.5	1.28	0.68-2.40	0.4	0.77	0.51-1.15	0.2	1.34	0.88-2.12	0.2
*Location based on final postcode:*													
Regional	401	1			1			1			1		
Metropolitan	599	1.47	0.72-3.001	0.3	2.11	0.81-5.44	0.1	1.51	0.96–2.38	0.08	1.62	1.0–2.6	0.08
*Years of smoking:*													
Less than or equal to 20 years	291	-	-	-	1			-	-	-	1		
More than 20 years	304				1.80	0.96-3.36	0.07				1.37	0.88–2.12	0.2
*Goodness of fit test for model*				0.390			0.191			0.236			0.678
(*p*<0.05 indicates poor fit)													

### Help-seeking behaviour

In contrast with the qualitative findings which suggested a low confidence, the majority of the population sample was very or fairly confident they would notice a symptom of lung cancer (59 %). Confidence did not differ across age, sex, SES, geographic or smoking status yet was dependent upon ever having being diagnosed with cancer in the past or having a close/friend or relative who had been diagnosed with a cancer (unspecific) (AOR 1.65; 95 % CI 1.09–2.48). The majority of participants (78 %) also reported they would see a doctor within a week if they had a symptom they thought might be associated with lung cancer, and there was no difference across demographic groups. There was however a difference in terms of telling family/partner, with ever smokers half as likely to tell a family member if they had any symptoms they thought might be associated with lung cancer (AOR 0.54; 95 % CI:0.37–0.81).

## Discussion

Across both qualitative and quantitative findings, identification of symptoms and risk factors was consistent, yet where the quantitative data provided correlations, the qualitative responses helped to explain these associations. For example, smoking as a risk factor for lung cancer was noted in the quantitative findings and qualitative findings, however some of the smoking groups either avoided mentioned smoking as a risk factor or they down-played the risk of smoking in discussing the risk (Table 4 matrix). Similarly, under-recognition of the risk of second-hand smoke, particularly in current smokers was observed in the qualitative data and confirmed in the quantitative analysis. These findings might suggest a low perceived susceptibility of lung cancer amongst smokers.

Recognition of symptoms was similar across the qualitative and quantitative findings with most of the sample recognising symptoms of haemoptysis and dyspnoea yet some confusion between emphysema and lung cancer observed in the focus groups study might explain the high recognition of dyspnoea in the survey findings. We had expected some variation in symptom recognition based on smoking status however the quantitative component indicated that current or former smokers were no more or less likely to recognise symptoms. It is perhaps unsurprising however that knowledge of symptoms was lower in the older age group. Previous studies indicate that older people are more likely to attribute symptoms to either ageing or co-morbidities [11] and as the risk of co-morbidity increases with age, difficulty in differentiating symptoms is also likely to increase. Similarly lower recognition of symptoms has been found in other studies, suggesting that men have a lower cancer awareness, poorer knowledge of cancer warning signs, less frequent contact with doctors and are thus more likely to delay in symptom presentation [33]. Greater confidence in recognising symptoms was associated with knowing someone who had been diagnosed with any type of cancer.

The ability to recognise a sentinel symptom for lung cancer appears high compared with findings in the UK [25]. However our qualitative study suggested that while symptom knowledge may appear high, many people would delay seeking help for symptoms. A low self-perceived risk and ‘wait and see’ attitude was prevalent across groups despite belief in both study components that if the person had symptom suggestive of lung cancer they would seek help promptly. These findings suggest that immediate help-seeking behaviours would not occur unless the symptom was considered serious, such as coughing up blood. These findings are consistent with evidence across cancer types, suggesting many patients may underestimate the urgency, attribute mild symptoms to common ailments or believe symptoms will go away [14]. This assumption that only major symptoms warrant medical attention has implications for prompting earlier diagnosis. While mild symptoms may be more likely to be ignored, they may be more common in initial stages of the disease [19] when surgical resection may offer better survival outcomes [5]. Other potential factors for delay in seeking medical help mentioned in the focus groups include difficulty in accessing a GP, particularly for people living in regional and rural locations [34]. Current and former smokers also expressed fear of judgement or blame as reasons for currently delaying GP visits. Unsurprisingly, reluctance to seek medical attention due to stigma around smoking has been described elsewhere as a reason people at risk of lung cancer delay presentation [17, 35]. There is therefore an important role for family and extended social networks in supporting these individuals to act upon their symptoms [36] and a critical role for health professionals in taking a no blame approach when interacting with current and former smokers.

This study is unique in that it considers evidence from both qualitative and quantitative studies, providing a richer understanding of the current community awareness of lung cancer. Without the qualitative findings the evidence would suggest people living in NSW have a reasonable understanding of lung cancer symptoms. However, the qualitative findings reveal that despite awareness of cancer symptoms and risks, high-risk individuals have poor perceptions about their personal risk and the seriousness of sentinel symptoms. While denial was high amongst current smokers, former heavy smokers might also have a false sense of security, with qualitative findings suggesting that public health messages about the benefits of quitting may have been interpreted as implying that the risk of lung cancer disappears on quitting.

In an attempt to reduce patient related delays to diagnosis, public health approaches which challenge poor perceptions of personal risk may have a significant potential to improve lung cancer outcomes for those most at risk. Potential public health approaches include the implementation of public education campaigns for the general population which outline key signs and symptoms of lung cancer. This is supported by the evaluation of a large-scale public awareness campaign in the UK which demonstrated an improvement in both knowledge of symptoms and lung cancer stage distribution following a sentinel symptom awareness campaign [10, 37]. In addition, complimentary public health messages to target individuals at high-risk of developing lung cancer would be beneficial given the disparity in knowledge and perceived risk. However, the fears of stigma indicate that health professionals interacting with current and former smokers would need to be well prepared to provide a safe and non-blaming environment for those presenting with symptoms suggestive of lung cancer.

The limitations of this study are that investigating population awareness of lung cancer signs and symptoms relied on cross-sectional data and hypothetical scenarios to explore help-seeking behaviours and to model symptoms. Longitudinal studies or case studies with lung cancer patients and assessment of disease staging would be needed to verify findings. In terms of sampling, the use of RDD sampling frame will have resulted in exclusion of mobile phone-only households which may have an effect on selection bias. We sought to minimise this through higher sampling of ever smokers given current smokers have a higher tendency to live in mobile-only households in Australia. The qualitative data may also have been limited in that the researchers were not present at all of the focus group sessions and focus group participants were not recontacted to validate comments made during the group discussion. The results may be ungeneralisable given the potential differences in exposure to messages relating to lung cancer.

## Conclusion

Drawing on insights from qualitative and quantitative sources, this study found that while awareness of lung cancer symptoms and causative factors may seem reasonably well understood in the community, perceived susceptibility is low, particularly amongst those most at risk. A lack of urgency in help-seeking for symptoms considered mild and therefore trivial, together with smoking-related barriers such as stigmatism and nihilism, may contribute to delay in diagnosis and poorer outcomes.

## Availability of data and materials

Quantitative datasets and other material used in this article can be accessed by request to the lead author. Qualitative data is not available for public use to maintain the privacy of participants.

## Abbreviations

Ex-S: previous smoker
NS: non smoker
S: current smoker
